# Objective Measures of Immediate “Energizing” Effect of Light: Studies Review and Data Analysis

**DOI:** 10.3390/clockssleep4040038

**Published:** 2022-09-26

**Authors:** Konstantin V. Danilenko

**Affiliations:** Institute of Neurosciences and Medicine, 630117 Novosibirsk, Russia; kvdani@mail.ru

**Keywords:** healthy subjects, light, immediate effect, physiological measures

## Abstract

While the energizing effect of light has been known since the early years of light therapy, its reliable detection using objective measures is still not well-established. This review aims to ascertain the immediate energizing effect of light and determine its best indicators. Sixty-four articles published before July 2022 were included in the review. The articles described 72 (sub-)studies performed in healthy individuals. Fourteen measures were analyzed. The analysis showed that light causes an energizing effect that can be best documented by measuring core (rectal) body temperature: the proportion of the studies revealing increasing, unchanging, and decreasing rectal temperature was 13/6/1. The second most suitable indicator was heart rate (10/22/1), which showed concordant changes with rectal temperature (a trend, seven mutual studies). There is no evidence from the reviewed articles that oxygen consumption, skin conductance, blood pressure, heart rate variability, non-rectal inner temperature (combined digestive, tympanic, and oral), skin temperature, or cortisol levels can provide light effect detection. Four other measures were found to be unsuitable as well but with less certainty due to the low number of studies (≤3): skin blood flow, noradrenaline, salivary alpha-amylase, and thyroid-stimulating hormone levels. On the other hand, light exposure had a noticeable effect on sympathetic nerve activity measured using microneurography; however, this measure can be accepted as a marker only tentatively as it was employed in a single study. The analysis took into account three factors—study limitation in design/analysis, use of light in day- or nighttime, and relative brightness of the light stimulus—that were found to significantly influence some of the analyzed variables. The review indicates that the energizing effect of light in humans can be reliably detected using rectal temperature and heart rate.

## 1. Introduction

From the early years of the use of light therapy in the treatment of seasonal affective disorder, an immediate “energizing” effect of light has been clinically noted (the term was first coined by Lewy and Sack in 1986 [1]). This effect was documented experimentally, for example, in a study by Danilenko et al. [2], revealing subjectively increased energy levels during exposure to a 4 h intense cool light compared to a dimmer red light. While energy levels were psychometrically estimated in only few light studies (e.g., [3]), topics of alertness, contextually close to “energy”, have been extensively examined. There is abundant evidence that alertness increases during bright light exposure based on both subjective estimates and EEG (electroencephalography) (reviewed in [4]).

The most obvious approach to objectively measure the energizing effect would be to link it to the change in energy expenditure by estimating oxygen consumption (a ‘semi-direct’ measure of overall metabolic rate). This indeed increased both in winter depressives and healthy controls following a week of bright light therapy [5]. However, the concrete physiological substrate for the energizing effect has still not been determined. Sympathetic–adrenal and energy-regulating systems have been proposed to be underlying the energizing effect in connection to winter depression [6].

**Figure 1:**
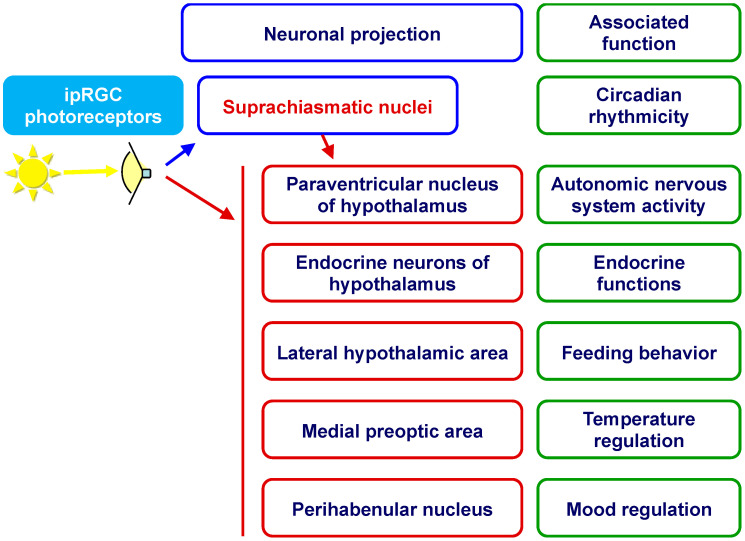
The ipRPG projections to neuronal centers of mammalian brain with different associated functions. Light influence may be indirect (via suprachiasmatic nuclei) or direct. ipRPG—intrinsically photosensitive retinal ganglion cells. Figure adapted and simplified from [13], updated according to data presented in [8,9].

**Figure 2:**
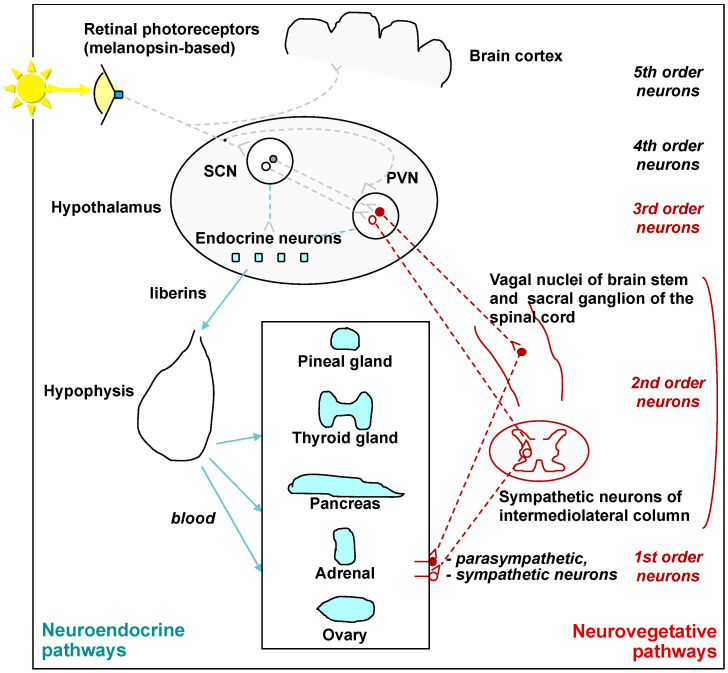
Light, endocrine system, and autonomic nervous system anatomical interrelation. The data are based partly on results of labeled viral transsynaptic retrograde tracing in rodents: after injection of the labeled virus to a peripheral gland, the tracer was consecutively found in neurons indicated as the 1st-to-4th order neurons [10,11,12]. SCN—suprachiasmatic nuclei, PVN—paraventricular nucleus. Reprinted from [14].

Light, acting via the eyes, targets various brain structures [7,8], many of which play a role in energy regulation (Figure 1). Light perception involves intrinsically photosensitive melanopsin-expressing retinal ganglion cells (ipRGCs), which are sensitive to blue light and have been proven to mediate effects of light on various physiological functions [7]. The importance of specific wavelengths of light for metabolic effects is discussed in a review by Fleury and coauthors [9]. The neuroendocrine and neurovegetative pathways to the periphery have been well-documented in studies on rodents [10,11,12] (Figure 2). The immediate energizing effect of light could therefore engage various processes and could be probed utilizing the following measures (the majority of which are mentioned in [13]):

- Total energy expenditure/metabolism (by oxygen consumption);
- Autonomic nervous system (ANS) activity:
  − Sympathetic nerve activity (by microneurography);
  − Galvanic skin conductance;
  − Skin blood flow/perfusion;
  − Salivary alpha-amylase;
  − (Nor)adrenaline concentration;
  − Blood pressure;
  − Heart rate, heart rate variability;
  − Pupillary light response;
- Thermogenesis (regulated in part by ANS; body temperature);
- Endocrine system activity (cortisol and thyroid hormone concentrations).

Some of these measures have been used for a long time; others were implemented only recently; some have certain limitations. For example, skin electrical conductance depends on the activity of skin sympathetic nerves controlling the sweat glands. It elevates as skin moisture increases and with a shift in electrolyte levels that occurs during states of emotional arousal [13]. Relatively high variability is also characteristic for blood pressure and heart rate indices. Direct assessment of sympathetic nerve activity is possible with microneurography by measuring the rate of electrical impulses at the nerve [15]. Although the technique is almost painless for the subject, precise insertion of the thin tungsten electrode in an efferent motor fiber of the nerve trunk demands skill and likely several tries; therefore, the method is not very widespread.

Assessment of cortisol (and some other hormones—melatonin, testosterone, and dehydroepiandrosterone) is possible in saliva, where their levels reflect their concentration in blood sufficiently well [16]. Sampling saliva is more feasible than taking blood or urine, and the measurement is performed using routine biochemical techniques. Recently, secretion of the salivary enzyme alpha-amylase was introduced as a measure of ANS activity [17]. The secretion of alpha-amylase is 60–75% dependent on noradrenergic neural activity [18] and, compared to cortisol, increases faster in response to stress. Not all analytes are detectable throughout a 24 h period in biofluids: melatonin is secreted only at night, for ca. 11.5 h (see melatonin profiles in [19]).

Physiological reactions to light may differ between unhealthy individuals and controls. For example, changes in noradrenaline urinary excretion had opposite trends in migraineur and control subjects [20], and heart rate variability behaved differently in winter depressives [21] or in individuals having symptoms of depression or anxiety [22] compared to controls. Therefore, this review is restricted to data from healthy subjects, as the goal of this review is not only to ascertain the energizing effect of light but also to determine the best indicators of this effect in a standard case. The review should narrow the existing knowledge gap about which biomarkers can reliably measure the effects of light on physiology and behavior [23].

## 2. Methods

This review is an extension of the previous scoping review made for the SLTBR meeting in 2017 [24] using the author’s database of publications and a literature search of the PubMed library. The author’s database was updated using citation sources (references in recently published articles and article citations by scholar.google.com). The review did not consider (1) studies on unhealthy subjects or case studies; (2) non peer-reviewed publications in the form of abstracts or proceedings; (3) combined interventions (e.g., light + ambient temperature) unless the light effect(s) could be separated for the analysis; (4) low-intensity light intervention (which is unable to suppress melatonin secretion); (5) if the effects measured far outweigh the intervention time; (6) psychometry and performance tests, due to their relative subjectivity; (7) EEG measures, due to their nonspecificity to the energizing effect; (8) melatonin levels, due to their nonspecificity to the energizing effect and close-to-zero values during daytime. Furthermore, the immediate effects of light on psychometric and EEG indices and melatonin are well-known and have been reviewed elsewhere [25,26].

Studies exploiting pupillary light response were also excluded, as pupil diameter diminishes rapidly during the first 1–2 min of light exposure and does not change significantly afterwards (e.g., [27]), making it not useful for long-term monitoring of the light effects.

## 3. Studies Review

Twelve measures were considered in the review: (1) oxygen consumption, (2) sympathetic nerve activity, (3) skin conductance (SC), (4) skin blood flow (SBF), (5) noradrenaline levels, (6) blood pressure (BP), (7) heart rate (HR), (8) heart rate variability (HRV), (9) body temperature, (10) salivary alpha-amylase activity, (11) cortisol levels, and (12) thyroid-stimulating hormone (TSH) levels.

In total, 64 articles were included in the review; they were all published before July 2022. Their data are presented in 3 tables made up of 62 entries (in two cases, two articles that reported data on the same study were combined: Scheer et al. [28,29]; Lok et al. [30,31]. Table 1 comprises studies that explored at least 2 of the 12 measures (with 2 exceptions; 36 entries). Table 2 summarizes studies measuring body temperature only (16 entries). Table 3 reports studies monitoring cortisol levels only (with 2 exceptions; 10 entries). The column “Subjects, design” in the tables indicates the actual number of subjects in the statistical analysis. The designations “within” and “between” signify crossover- and parallel-group design, respectively. “Within” design suggests a counterbalanced order (when subjects are equalized to get one or another light intervention first), unless indicated as not counterbalanced (“NCB”). In the NCB case, the comparative arm usually preceded the active (light intervention) arm of the study, which limits the interpretation of the obtained results. The additional term “alternating” means that the same light intervention was used several times throughout the day (five studies: [28,32,33,34,35]).

Not included were two studies with simple design in which the absence of a comparator (e.g., dim light) made it impossible to differentiate the true effect of light from the natural dynamics of the indices (due to some variations over the day, prolonged rest, etc.): a study on HR, blood pressure, and urinary catecholamines excretion [20] and a study on HRV [36]. Two short-form reports were not included: an abstract by Badia et al. [37] on skin conductance and a proceedings by Beck-Friis et al. [38] on cortisol secretion (only four subjects and NCB crossover design).

**Table 1:** Immediate effect of bright light on heart rate (HR), heart rate variability (HRV), body temperature (T), and/or some other variables (oxygen consumption—OC, sympathetic nerve activity, skin conductance—SC, skin blood flow—SBF, catecholamines, blood pressure—BP, cortisol—Cor, and thyroid-stimulating hormone—TSH) in healthy subjects.

N	Study	Subjects, Design	Light, Posture, Data Acquisition	Effect			
				HR	HRV	Body T	Other
1.	Rechlin et al., 1995 [21]	18 (13 f, 5 m), within	1.5 h 2500 lx vs. <200 lx 6:00–7:30; supine; ECG before/after	= HR	↑ HF = VLF, LF		
2.	Saito et al., 1996 [15]	5 males, within	20 min 5000 lx vs. 500 lx (20 min pre and post 500 lx), between 10:00–12:00, BP continuously using a tonometric system	↑ HR			↑ sympathetic nerve activity = BP
3.	Leproult et al., 1997 [39]	17 males, within NCB	3 h 5000 lx (vs. light < 300 lx 2 wk prior), between 22:00–08:00 (individually); supine and awake for 37–39 h; pill-ingested telemetric T°, blood taken every 20 min			= digestive T	= Cor = TSH
4.	Ishimoto et al., 1998 [40]	4 males, within	3 h 2500 lx vs. 200 lx 10:00–13:00; sitting; esophageal, tympanic, and forehead skin T	= HR		= core T = forehead T	
5.	Scheer et al., 1999, 2004 [28,29]	17 (6 f, 11 m): 10 males: both—alternating	0 lx 20 min → indoor light 20 min, 5 times over 24 h, at daytime; supine 0 lx 20 min → 100 lx 10 min → 800 lx 10 min, 5 times over 39 h, including night (during middle and end of sleep); supine	↑ HR ↑ HR at night	= SDNN = SDNN		
6.	Sakabibara et al., 2000 [41]	12 females, within	1 h 5000 lx vs. dim 18:00–19:00, two successive days; 5 min measurement at 19:00 only		↑ LF = HF, LF/HF, HRV		
7.	Kim, Tokura, 2000 [42]	5 aged females, within	5 h 3000 lx vs. 50 lx 9:30–14:30; sitting; HR and BP using electronic sphygmomanometry	= HR		↓ ReT at 13:50–14:30	= BP **
8.	Leproult et al., 2001 [43]	8 males, within	3 h 2000–4500 lx vs. dim < 150 lx another day -at night/morning 5:00–8:00 or -in the afternoon 13:00–16:00 supine and awake for 24 h each of the 3 days; blood (plasma) taken every 15 min	= HR = HR			↑ Cor, = TSH = Cor, TSH
9.	Burgess et al., 2001 [44]	16 (8 f, 8 m), within NCB	4.5 h 3000 lx vs. <10 lx day prior, starting at ~19:00—2 h before the estimated time of melatonin secretion onset (pre-pulse < 10 lx); supine; 2 skin thermistors on soles; BP using ‘standard’ sphygmomanometry (3 readings at each 30 min time point); measurements starting 0.5 h after the pulse onset	= HR *	= %HF	↑ ReT in 3 h ↓ peripheral skin T	↑ sBP in 3.5 h = dBP (*n* = 12)
10.	Lavoie et al., 2003 [45]	14 (8 f, 6 m), within	4 h white 3000 lx vs. red < 15 lx 00:30–04:30; sitting; urine taken every ~2 h			↑ ReT in 2.5 h	= Cor
11.	Cajochen et al., 2005 [46]	9 males, within	2 h bright blue 460 nm vs. bright green 550 nm vs. dark, 21:00–23:00 (2 h pre-pulse 0 lx); supine; 8-site skin T using thermocouples; 20 min binned values	↑ HR in blue light in 1.5 h		↑ ReT in blue light in 1 h ↓ DPG at either light in 1 h	
12.	Rüger et al., 2006 [3]	Two studies 12 males: 12 males: each—within	4 h 5000 lx vs. <10 lx (pre- and post-pulse < 10 lx) –at 0:00–4:00 or –at 12:00–16:00 sitting; saliva sampled every 30 min	↑ HR = HR		↑ ReT = ReT	= Cor = Cor
13.	Yokoi et al., 2006 [47]	8 males, within	7.4 h 2800 lx vs. 120 lx 21:10–4:30 (pre-pulse 120 lx from 19:30); supine; measurements from 21:10; ReT—*n* = 7, BP using photoplethysmography (*n* = 6)	↑ HR	= %LF, %HF, LF/HF	↑ ReT in 3 h	↑ sBP in 4 h = dBP
14.	**Schäfer, Kratky, 2006** [48]	12 subjects, within	10 min 700 lx blue vs. green vs. red (15 min pre and post: 0 lx), after 21:00 on separate(?) days in winter		↓ very LF in blue		
15.	**Ishibashi et al., 2007** [49]	7 males, within	6.5 h 1000 lx, 3000 K vs. 5000 K vs. 6700 K 19:30–02:00 (1.5 h pre-pulse 10 lx); supine	= HR	= HF ↓ fractal-free HF at 6700 K		
16.	**An et al., 2009** [50]	12 males, within	20 min 67 lx, blue 458 nm vs. green 550 nm, sequentially (for 35 min), in the evening (before sleep) vs. 12 h apart, baseline 19 lx → 11 min pre-pulse < 1 lx; oral temperature before/after, HR and BP using photoplethysmography	= HR	↑ HF/(HF + LF) by blue vs. green at daytime	↑ oral T in blue vs. green in daytime and blue in evening	= BP **
17.	**Laufer et al., 2009** [51]	12 adults 66–84 y, within	8 min 300 lx, blue vs. red (6 min pre-pulse 0 lx) sequentially between 13:00–17:00, reverse sequence on another day; sitting		↑ HF, ↓ LF/HF at blue		
18.	Ishibashi et al., 2010 [52]	10 males, within	2.5 h 5000 lx vs. 30 lx 21:30–24:00 (0.5 h pre-pulse 650 lx); semi-supine; 7-site skin T° by thermistors, BP using electronic sphygmomanometry once at 21:15 and at 23:45, oxyspirography at 23:55 (while moving)	= HR	↓ LF = HF, LF/(LF + HF)	= ReT, DPG ↑ proximal and distal skin T in the last 30 min	= OC = BP
19.	Choi et al., 2011 [22]	55 subjects, within	5 min white 49.5 lx vs. blue 0.04 lx vs. red 0.4 lx (10 min pre- and post- 0 lx), each of 3 in any of 4 time slots between 09:00–11:00 and 14:00–16:00 for two successive days; sitting; 5 min ECG before and after each pulse	= HR	↑ LF/HF in white and red vs. blue = RMSSD, HF		
20.	**Litscher et al., 2013** [53]	7 (5 f, 2 m), within	10 min 140 lx, blue 461 nm vs. red 621 nm between 9:00–11:00 on a single day; lying; 2-site skin T using infrared camera	↓ HR	↓ SDNN * = LF/HF	= forehead T * ↓ nose T *	
21.	Smolders et al., 2012 [54]	32 (13 f, 19 m), within ‘mixed’	1 h 650 lx vs. 230 lx (0.5 h pre-pulse 120 lx) at 9:30, 11:30, 13:30, and/or 15:30 on separate days (each subject participated in at least 2 of the 4 arms)	↑ HR	= LF, HF ↑ LF/HF at 45 min		
22.	Smolders, de Kort, 2014 [55]	28 (16 f, 12 m), within	30 min 1000 lx vs. 200 lx (45 min pre-pulse 92 lx) on separate days in the same timeslot between 9:00–18:00; sitting	= HR	= LF/HF		↑ SC
23.	Huiberts et al., 2016 [56]	39 (28 f, 11 m), within/ between	1 h 1700 lx vs. 600 lx vs. 165 lx (0.5 h pre-pulse 120 lx) –at 09:00 (*n* = 18) or –at 15:45 (*n* = 21) sitting; ECG (*n* = 34), two electrodermal electrodes on hand (*n* = ?), BP using photoplethysmography (*n* = 35)	= HR ↑ HR 1700 lx			= SC, sBP = SC, sBP
24.	**Yuda et al., 2016** [57]	10 (1 f, 9 m), within	6 min blue 10 lx vs. green 71 lx vs. red 39 lx (3 min pre- and post- 0 lx) between 08:30–13:00 on a single day; supine	= HR	↓ HF with blue = LF, LF/HF		
25.	**Canazei et al., 2017** [58]	31 (18 f, 13 m), within	4 h 150 lx, 4667 K vs. 3366 K vs. 2166 K color T°, 0:00–4:00; sitting	↑ HR * for 2.5 h at 4667 K	= SDNN, LF ↓ RMSSD *, HF * ↑ LF/HF * at 4667 K		
26.	Ivanova et al., 2017 [59]	10 females, within	30 min white 4300 lx vs. red 250 lx between 9:00–10:00 after coming into the lab, 25 min pre and post < 100 lx; sitting; 5 min pulsemetry + oxyspirography, blood (serum)—3 times	= HR			= OC = Cor = alpha-amylase
27.	Te Kulve et al., 2017 [60]	19 females, within	4.5 h 1200 lx vs. 5 lx, 08:30–13:00 (0.5 h pre-pulse 250 lx); every 1.5 h free moving 15′ + supine 75′; pill-ingested T°, 26-site skin T°; oxyspirography, laser doppler flowmetry—continuously, while supine; BP using electronic sphygmomanometry—3 times; blood (plasma) taken at pre-pulse and every 1.5 h	= HR		↓ digestive T° ↓ proximal skin T° = distal skin T° ↑ DPG	= OC, SBF, BP = dopamine = noradrenaline ↓ adrenaline ↓ Cor *
28.	**Te Kulve et al., 2018** [61]	16 females, within	4.5 h 55 lx 6500 K vs. 55 lx 2700 K, 08:30–13:00 (pre-pulse 5 lx); the remaining is the same as in the cell above	= HR		= digestive T° * = skin T°, DPG	= OC, SBF, BP = Cor
29.	Te Kulve et al., 2019 [62]	12 females, within	1 h 750 lx vs. 5 lx 22:30–23:30 (pre- and post-pulse for 1 h); sitting; telemetric pill-ingested T°, 26-site skin T°, oxyspirography, laser doppler flowmetry	= HR		= digestive T° = DPG	= OC = SBF
30.	Smolders et al., 2018 [63]	60 (41 f, 19 m), within/between	1 h pulse of 20 various intensities between 20–2000 lux (0.5 h pre-pulse 100 lx) at 9:00 or 11:00 vs. 13:00 or 15:00 for two separate days; sitting; 2 electrodermal electrodes on hand	= HR			= skin conductance
31.	Lok et al., 2019 [64]	10 (5 f, 5 m), within	1.5 h 2000 lx vs. 10 lx 14:30–16:00 (2.5 h pre-pulse 10 lx); sitting(?); under-tongue telemetric T pill, 10-site skin T	= HR		= under-tongue T ↑ hand skin T, DPG	
32.	Prayag et al., 2019 [27]	28 males, within	50 min pulse: blue-enriched 298 melanopic lx vs. red 80 mlx for the 1st min adding more centric vs. less centric white light for the next 49 min: 494 mlx vs. 276 mlx vs. 620 mlx vs. 402 mlx; 10 min pre-pulse < 5 lx = four 1 h arms between 19:00–23:00 (18:00–19:—<5 lx); sitting; ECG, 2-site skin T; analysis during min −1, 1, 2, 5, and 42	↑ HR for 4 lights altogether at min 2, 5, 42	= LF, HF, LF/HF; for between-light effects see paper	↑ DPG for 4 lights altogether at 5 and 42 min	
33.	Kompier et al., 2020 [65]	38 (19 f, 19 m), within	45 min cool 1000 lx vs. warm 100 lx (45 min pre-pulse either cool or warm) = 4 arms on separate days during the same daytime slots; sitting; four 5 min measurements (−13, 2, 17, and 32 min) analyzed. 14-site skin T, 2 electrodermal electrodes on hand	= HR	= ‘mean’ HRV	= skin T° = DRG	= skin conductance
34.	Kompier et al., 2021 [66]	23 (13 f, 10 m), within	45 min cool 1000 lx vs. cool 100 lx vs. warm 1000 lx vs. warm 100 lx (45 min pre-pulse warm 100 lx); the remaining is the same as in the cell above	= HR	= ‘mean’ HRV	= skin T° = DPG	= skin conductance
35.	Schmid et al., 2021 [67]	33 males, within	1.5 h light pulse melanopic 287 mW 8300 K vs. 114 mW 3000 K vs. 0.4 mW 2200 K 21:45–23:15 (pre- and post-pulse < 5 lx); sitting, allowed to move; 4-site skin T (*n* = 30), salivary cortisol at min 0 and 90 of light pulse			= DPG	= Cor
36.	Lok et al., 2022, 2022 [30,31]	8 males, within, between	13 h lights during three 18 h forced desynchrony days 1300 lx and 6 lx; sitting/moving; pill-ingested telemetric T (*n* = 8 subjects, crossover), 10-site skin T (*n* = 4 and *n* = 4, parallel groups), Cor in saliva sampled hourly (*n* = 7, crossover)			= digestive T° ↑ proximal skin T ↓ distal skin T	= Cor

References in bold—light wavelength studies. Body T column: includes rectal temperature (ReT), digestive, tympanic, oral, skin T, distal-to-proximal gradient (DPG) of skin T (↓ DPG is usually associated with ↑ ReT); sBP, dBP—systolic and diastolic blood pressure, respectively; N—number; NCB—not counterbalanced; lx—lux; K—Kelvin. *—the effect could disappear (or emerge) if the data series were compared after adjusting for the difference in the initial (pre-pulse) values; **—the increase in BP reported in two studies was recategorized as “no effect” (see explanation in Section 3.6).

**Table 2:** Immediate effect of bright light on body temperature—including rectal temperature (ReT), tympanic T, and distal-to-proximal gradient (DPG, a derivate of skin T) in healthy subjects.

N	Study	Subjects, Design	Light, Posture, Data Acquisition	Effect
1.	Dijk et al., 1991 [68]	7 males: 8 (3 f, 5 m): both—within	3 h 2500 lx vs. 6 lx 21:00–24:00; sitting from 19:30 3 h 2500 lx vs. <1 lx (goggles) 20:30–23:30; sitting(?) pre-pulse(?)	↑ ReT ↑ ReT
2.	Badia et al., 1991 [32]	16, alternating; 8, alternating; 19, between	bright 5000 lx vs. dim 50 lx 1.5 h bright /1.5 h dim × 3 blocks 0:00–9:00 1.5 h bright /1.5 h dim × 2 blocks 13:00–19:00 9 h bright (*n* = 10) vs. dim (*n* = 9) 21:45–7:45 sitting; tympanic T every 30 min	↑ T (except 0:00–1:30)= tympanic T ↑ tympanic T
3.	Myers, Badia, 1993 [69]	15 males, within	2 h 5000 vs. 1000 vs. 500 vs. 50 lx 21:00–23:00 (pre-pulse 50(?) lx); sitting; tympanic T every 15 min from 19:00	↑ tympanic T (in ~1 h; 5000 = 1000 = 500 > 50 lx)
4.	Kim, Tokura, 1995 [70]	7 females, within	8 h 4000 lx vs. 10 lx 10:00–18:00 (light before 10:00 not controlled); sitting(?); measurements starting at 10:00	= ReT
5.	Morita, Tokura, 1996 [71]	5 males, within	5 h 1000 lx 6500 K vs. 1000 lx 3000 K vs. 50 lx dim 21:00–2:00 (2 h pre-pulse 50 lx); sitting	↑ ReT (each bright vs. dim)
6.	Morita et al., 1997 [72]	4 males, within	5 h blue 435 nm vs. green 545 nm vs. red 610 nm, each at 2500 lx vs. 1000 lx vs. 50 lx incandescent light 4:00–9:00 (pre-pulse sleep 22:00–04:00), 7 arms in total; posture(?)	↑ ReT at green 2500 lx
7.	Kräuchi et al., 1997 [73]	9 males, within	3 h 5000 lx vs. <10 lx 21:00–24:00 (7 h pre-pulse < 10 lx); supine	↑ ReT (to the end of the pulse)
8.	Aizawa, Tokura, 1997 [74]	9 (7 f, 2 m), within	8.5 h 4000 lx vs. 100 lx 9:30–18:00, pre-pulse light not controlled; posture(?); tympanic T every min	↓ tympanic T 16:45–18:00
9.	Aizawa, Tokura, 1998 [75]	5 females, within	4 h 5500 lx vs. 150 lx 9:00–13:00 (post 150 lx until 16:00); posture(?); tympanic T 10:00–16:00	↓ tympanic T 11:00-onwards
10.	Foret et al., 1998 [76]	8 males, within	4 h 700–1000 lx vs. 50 lx, 20:00–24:00, (pre-pulse light—“from windows”); sitting	= ReT *
11.	Park, Tokura, 1998 [77]	8 females, within	13 h pulse 5000 lx vs. 200 lx 6:30–19:30 (until 06:00—sleep); sitting	= ReT
12.	Zhang, Tokura, 1999 [78]	9 females, within	6 h 5000 lx vs. 50 lx, 6:00–12:00 30 (until 06:00—sleep); sitting	= ReT
13.	Cajochen et al., 2000 [79]	13 (at least 12 are males), between	6.5 h ~3190 lx (*n* = 7) or ~23 lx (*n* = 6) at night (centered 3.5 h before the expected ReT minimum; pre-pulse 3 lx); supine	↑ ReT (in 1.5 h)
14.	Rüger et al., 2003 [80]	7 males, within	4 h 5000 lx vs. <10 lx, 0:00–4:00 (17 h pre-pulse 10 lx); sitting	↑ ReT
15.	Sato et al., 2005 [81]	9 (4 f, 5 m), within	2 h 2500 lx 6480 K vs. 2500 lx 3150 K vs. <50 lx in the morning (2 h after ReT minimum) after sleep; posture(?)	↑ ReT (at 6480 K vs. both 3150 K and dim)
16.	Lok et al., 2018 [35]	50 (25 f, 25 m), between and within (alternating)	1 h pulse of 24 or 74 or 222 or 666 or 2000 lx (*n* = 10 in each group) at 9:00, 11:30, 14:00, and 16:30 (1.5 h pre-pulse < 10 lx); sitting; 6-site skin T	↑ DPG

N—number; Lx—lux; K—Kelvin. *—the effect could be categorized as “↑ ReT” if the rectal T data series were compared after adjusting for the difference in the initial (pre-pulse) values.

**Table 3:** Immediate effect of bright light on cortisol (Cor) and alpha-amylase in healthy subjects.

N	Study	Subjects, Design	Light, Posture, Data Acquisition	Effect
1.	Petterborg et al., 1991 [82]	13 (7 f, 6 m), within	15 min 1500 lx vs. <200 lx at 22:00 (*n* = 5) or 23:00 (*n* = 8) (pre and post < 200 lx); sitting; blood (serum) taken at different times between 20:00–24:00 or 20:00–08:00	= Cor
2.	McIntyre et al., 1992 [83]	6 (2 f, 4 m), within	3 h 600 lx vs. <10 lx at 0:00–3:00 (2 h pre-pulse 10 lx); sitting; blood (plasma) taken hourly 23:00–05:00	= Cor
3.	Kostoglou-Athanassiou et al., 1998 [84]	10 males, within	6 h 5500 lx vs. 500 lx at 20:00–02:00 (pre-pulse light not controlled); blood (serum) taken at 16:00 and every 2 h from 20:00	↓ Cor *
4.	Scheer, Buijs, 1999 [85]	14 males, within NCB	1 h 800 lx vs. 0 lx the day prior –at ~07:00 (after awakening) –at ~23:00 (before sleep; pre-pulse(?) lx; *n* = 12) supine; saliva taken every 20 min during pulse	↑ Cor at 20, 40 min = Cor
5.	**Lockley et al., 2006** [86]	13 subjects, between	6.5 h blue (460 nm, *n* = 6) or red (555 nm, *n* = 7) light of equal photon density starting at ~23:00 h, ~72 h pre-pulse < 2 lx; supine; pupils dilated; blood (plasma) taken every 20–30 min, Cor values expressed as a percentage of the values at corresponding clock times on the previous day	= Cor
6.	Jung et al., 2010 [87]	20 (5 f, 15 m) 8 subjects: 5 subjects: 7 subjects: within NCB, between	6.7 h 10,000 lx (vs. 3 lx the day prior) –from ~02:00 onwards; –from ~08:00 onwards; –dim 3 lx both days; ~48 h pre-pulse 3 lx; sitting; blood (plasma) taken every 30 min	↓ Cor in ~2.5 h ↓ Cor in ~0.5 h (= Cor)
7.	Figueiro, Rea, 2010 [33]	12 (8 m, 4 f), within, alternating	1 h blue 40 lx vs. red 40 lx vs. <3 lx, each—on separate days every 4 h for 7 times over 27 h, <3 lx the rest of the day; 2 h sitting (before and during pulse); saliva sampled at the beginning and at the end of each pulse	= Cor = alpha-amylase
8.	Sahin et al., 2014 [34]	13 (7 f, 6 m), within, alternating	2 h white 361 lx vs. red 213 lx vs. dim < 5 lx, each—on separate days at 07:00, 11:00, and 15:00, <5 lx the rest of the day; saliva sampled at pulse hours 0, 1, and 2	= Cor = alpha-amylase
9.	**Danilenko, Sergeeva, 2015** [88]	16 females, within	45 min cool white 1300 lx vs. red 1100 lx in the morning after coming to lab in dark glasses; pre-pulse sitting for 5 min; blood (serum) taken at pulse min 0, 22, and 44	= Cor
10.	Petrowsky et al., 2019 [89]	Two studies 30 males: **23 males:** within	1 h 05:00–06:00 (right after awakening) –bright 414 lx vs. dim < 2 lx –blue 201 lx vs. green 806 lx vs. red 235 lx equal photon density saliva every 15 min 7 times	↑ Cor at 45–90 min ↑ Cor by blue and green at 60–75 min

References in bold—light wavelength studies; N—number; NCB—not counterbalanced; *—there may be no effect if the cortisol data series were compared after adjusting for the difference in the initial (pre-pulse) values.

Studies varied greatly in terms of utilized light sources, characteristics of the applied light, and comprehensiveness of the light information provided in the study reports (Supplementary File S1). Eleven studies were ‘light wavelength’ studies rather than ‘light intensity’ studies, meaning the stimuli were equal or similar by irradiance, power, or photon density, and there was no dim pulse control (Table 1: [48,49,50,51,53,57,58,61]; Table 3: [86,88], and study II in [89]). In view of melanopsin photoreception in the eye (see Section 1), the active light in these studies (blue or cool white) was higher in melanopic lux (mlx) [90] than comparative light of other color temperatures (red or green or warm white).

Four studies investigated the effects of light applied at quite different times on separate days for the same subjects: for example, comparing daytime and nighttime exposure [3,43,56,85]. In these cases, the light effects were considered separately in the analysis.

Active and comparative interventions were usually performed on different days separated by an intervening period (unless otherwise specified; Table 1, Table 2 and Table 3). The subject’s posture/movement status is reported in the Tables because it can influence some variables, including heart rate and body temperature, for which it is a recognized confounder. The majority of studies employed similar techniques for data acquisition. Rectal temperature (ReT) was measured using a thermocouple probe inserted into the rectum, skin temperature was measured using iButtons, and HR and HRV were measured using ECG (unless otherwise specified). All data were recorded continuously (unless otherwise specified).

### 3.1. Oxygen Consumption

Five studies reported oxygen consumption [52,59,60,61,62] (Table 1). Oxygen consumption was measured using oxyspirography via a ventilation mask connected to a gas analyzer. Although oxygen consumption was not measured repeatedly in one of these studies (only at the end of each light exposure, which limits the reliability of the results) [52], other studies also did not detect an immediate effect of brighter light on energy expenditure.

### 3.2. Sympathetic Nerve Activity

One early study measured sympathetic nerve activity using microneurography of the peroneal nerve [15] (Table 1). Bright light was found to increase the electrical burst rate registered from the efferent motor fibers along with an increase in heart rate.

### 3.3. Skin Conductance 

The measurement of skin conductance on the hand, or electrodermal activity (EDA), was reported in five articles of the same investigative group [55,56,63,65,66] (Table 1). Only one of these studies revealed a differential effect of light—brighter light increased skin conductance, which evidenced greater sympathetic tone in the test subjects.

### 3.4. Skin Blood Flow

Skin blood flow was measured using laser doppler flowmetry in three studies, all done by the same authors [60,61,62] (Table 1). The method relies on the passage of incident light through the skin followed by detection of a per-heart-beat change in reflected red light. The probes were attached to the ventral surface of the hand and the underarm and connected to a specialized device. No differential effect of bright vs. dim light was found.

### 3.5. Noradrenaline

A single light intervention study measuring catecholamines was included [60] (Table 1). It reported no effect of daytime light exposure on noradrenaline and dopamine and an unexpected decrease in adrenaline.

### 3.6. Blood Pressure

Nine articles reported measurement of arterial blood pressure (BP; 10 reported effects; Table 1). Five studies used standard sphygmomanometry with a cuff on the upper arm. The readings at each time point were made either once [52], three times [44,60,61], or were not specified [42]. Three studies used photoplethysmography that automatically calculates BP from volumetric pulse waveforms every second. In two of these studies, the sensor was attached to the finger [47,50], and in one, it was attached to the earlobe [56]. One early study used a tonometric system that combined a cuff on the upper arm and sensors on the wrist, which also allowed continuous measurement of BP [15].

A significant effect of light on BP was detected in only two studies, both using a prolonged 4.5 or 7.4 h light pulse starting in the evening (before or around the time of melatonin secretion onset) [44,47]. Interestingly, in both studies, the increase in BP was documented only for systolic (but not diastolic) BP, and this increase was registered only after 3.5–4 h from the start of light exposure, which suggests that the effect may be melatonin-dependent or could represent a circadian phase shift. In any case, this effect cannot be considered immediate. There were two other studies reporting an increase of BP during light exposure [42,50]. However, for this review, the results were classified as “no effect”, as they become not significant after adjustment of the data series for the difference in the initial (pre-pulse) BP values in active and comparator light conditions.

### 3.7. Heart Rate

Heart rate studies are summarized in Table 1. In total, 29 studies were identified. The majority of them showed no change or an increase in HR during the light presentation. Only one study showed a HR decrease. In this study [53], effects of blue and red light on HR were not directly compared by rANOVA or other statistics. Therefore, the reliability of the conclusion cannot be judged. In HR studies, posture had no influence on light-effects distribution; i.e., there was a similar ratio of yes/no effects on HR between the studies in which the subjects were sitting or supine during light intervention.

### 3.8. Heart Rate Variability

Data on heart rate variability (HRV) are summarized in Table 1 (19 studies, 20 reported effects). Commonly, HRV was assessed based on the analysis of a 5 min ECG recording for the following indices: root mean square of successive inter-beat differences (or SDNN, ‘standard deviation of normal-to-normal’ intervals, ms), root mean square of successive differences in NN intervals (RMSSD, ms), a ‘power’ (ms^2^) in spectral bands of low frequency (LF, 0.04–0.15 Hz) and high frequency (HF, 0.15–0.4 Hz) [13,48]. The latter two metrics are obtained using a special Fourier spectral analysis. Empirically, greater sympathetic activity (or lower parasympathetic activity, or greater sympathovagal ratio) is associated with lower inter-beat variability (↓SDNN, ↓RMSSD), higher LF power (↑LF), lower HF power (↓HF), and/or higher LF/HF power ratio (or ↑LF/(LF + HF)) [91].

The results of the HRV studies were heterogeneous: all three possible effects—a shift of the sympathovagal balance towards activation of either the sympathetic system or parasympathetic system or no effect—were reported with almost equal frequency. This heterogeneity may rise from the experimental conditions. For example, many HRV studies (7 out of 20) were light wavelength studies investigating blue vs. non-blue light effects. In addition, several of the reviewed HRV studies had different limitations in the methodology. Namely, Sakabibara et al. [41] measured HRV only at the end of each of the two light pulses without recording the pre-pulse values. The crossover study by Burgess et al. [44] was not counterbalanced. Studies by Schäfer and Kratky [48] and Litscher et al. [53] did not compare the two light conditions (blue and non-blue light) using rANOVA or similar statistics and focused instead on the dynamics of HRV values within each light condition (“before–after”).

### 3.9. Body Temperature

Data on body temperature are summarized in Table 1 (20 studies) and Table 2 (16 studies). The body temperature (T) measurements were subdivided into inner and skin T. Inner T was most frequently measured using a rectal probe. Whenever this is done (18 studies), inner T is reported as rectal T and abbreviated as “ReT” in the tables. A minority of the studies used other approaches for the measurement of the inner T: tympanic (5 articles), oral (*n* = 2), and digestive (using either ingested telemetric pill, *n* = 5 or esophageal thermistor, *n* = 1). Skin T was measured either simultaneously with inner T (*n* = 9) or alone (*n* = 6). Skin temperatures of feet or hands (“distal”) and of proximal sites (“proximal”) were analyzed separately in most studies because they are known to change in opposite ways [92]. Therefore, if the reported values represented a sum of the distal and proximal temperatures (e.g., [42]; Table 2), they were not included in the review. In the majority of the ‘skin T’ studies, both distal and proximal skin temperatures were measured and the distal-to-proximal gradient [92] was additionally analyzed.

Many studies showed an elevation of inner T during light exposure. It is noteworthy that the manifestation of the effect usually required some time—from 0.5–1 h (e.g., [46]; Table 1) to 2–3 h or even longer (e.g., [42]; Table 2). Skin T can be seen to respond faster (e.g., within minutes [27]; Table 1) or at the same rate as rectal T (e.g., [46]; Table 1).

Relatively few studies had methodological limitations. Studies by Leproult et al. [39] and Burgess et al. [44] were not counterbalanced. In the study by Foret et al. [76] (Table 2), bright light was administered at either 20:00–24:00 or 4:00–8:00 on the first night (crossover). Dim light was, instead, always administered on the second night. In this case, a reliable comparison can only be made between 20:00–24:00 of the first night, because the rectal T values at 4:00–8:00 on the first night and the values on the second night were affected by the prior light exposure (due to a light after-effect and circadian phase shift, respectively) and therefore cannot be used for comparison. Additionally, it is possible that in the study by Burgess et al. [44] mentioned above plus two other studies [53,61] (Table 1), differences in body T values between two lighting conditions would have emerged if the observed temperature time series were compared after adjusting for the difference in the initial (pre-pulse) values.

Body temperature is sensitive to ambient temperature (e.g., [52]) and changes in posture [93]—it takes up to 1 h for the temperature to return to a steady state after postural change. In most studies, ambient temperature was controlled and subjects were constantly supine or sitting before and during light exposure. In two studies, subjects were allowed to move three times for 15 min during the 4.5 h light presentation [60,61] (Table 1), and in four studies the posture was not specified ([30,64] in Table 1; [70,72] in Table 2). 

### 3.10. Alpha-Amylase

Light effects on salivary α-amylase levels were investigated in three studies (Table 1 and Table 3). None of the studies showed any significant effect due to light. The applied light was probably of too low intensity in both absolute and relative values (light wavelength rather than intensity was the focus) [33,34] or too short in duration (30 min) [59] to elicit an effect.

### 3.11. Cortisol

The immediate effect of light on cortisol levels was reported in 19 articles (Table 1 and Table 3), with 24 reported effects in total. Four reports from three well-controlled studies [43,85,89] supported the expected stimulating effect of bright light on cortisol, 16 reports (from 13 studies) yielded a neutral result, and 4 reports (from 3 studies) pointed to a cortisol level decrease. However, it is unclear how significant the reported decrease is in those three studies. In two of them, the effect might not remain significant after adjusting the cortisol curves for the difference in the pre-pulse values ([60] in Table 1; [84] in Table 3). In the third study ([87] in Table 3; two reported effects), prolonged light exposure was used after the subject spent two days in very dim light. Hence, the suppressing effect of light in this study might be related to a “considerable reduction in endogenous circadian amplitude” [87].

### 3.12. Thyroid-Stimulating Hormone

The immediate effect of light on thyroid function was investigated in only three studies, published in two articles ([39,43] in Table 1). They examined the effect of light on thyroid-stimulating hormone (TSH); two were performed at night. No significant effect of light on TSH levels was found.

## 4. Data Synthesis and Analysis

### 4.1. Data Synthesis and Description

In order to gather from the reviewed articles whether the energizing effect of light was detected and by which measures it was detected, data synthesis and analysis was performed. In total, the 64 reviewed articles described 72 (sub-)studies that reported an effect of light on at least one of the 12 measures of interest (oxygen consumption, blood pressure, etc.; Table 4). The number of reported effects was not equal to the number of articles describing those effects since some articles combined two or three separate studies (5 articles included 11 reports) or reported on two effects measured at different times of the day (4 articles contained 8 separate reports) or, inversely, two different articles reported data from the same study (4 articles contained 2 reports).

Table 4 presents the overall number of reported effects of light on each measure, the number of these effects by result (energizing, no effect, or opposite to energizing), and the percentage of energizing effects for each measure. Effects of light on either HRV or skin T were categorized as ‘energizing’ or ‘opposite to energizing’ if at least one of the measurements showed a significant result (for example, if a change of skin T in at least one body site was significant). The effects on body temperature were calculated separately for inner T and skin T as they may behave differently and were not measured simultaneously in many studies. Inner T was further subdivided into rectal T and non-rectal T, as the distribution of the effect direction was notably different between them (Table 4). The percentage of reported energizing effects was the highest for sympathetic nerve activity (100%, 1 out of 1 report), followed by rectal T (65%, 13 out of 20 reports), inner T (50%), and then HRV, HR, non-rectal inner T, and skin T (27–35%) (Table 4).

### 4.2. Statistical Analysis

For the statistical analysis, the directions of light effects—energizing, no effect, or opposite to energizing—were coded as “1”, “0”, or “−1”, respectively. An acceptable primary approach to analyze binary (‘vote counting’) data in systematic review is a non-parametric sign test [94]. Analysis was performed using SPSS 21.0 software. According to the one-sample sign test, the energizing effect significantly outnumbered the opposite-to-energizing effect for rectal T (*p* = 0.002), overall inner T, and HR (*p* = 0.012); for other measures, the result was not significant (Table 4).

This result indicates that rectal T is better suited for the detection of the energizing effect of light compared to the other measures. However, as the investigated measures were used in different studies, differences in experimental conditions may have potentially influenced the results. For example, in all studies that monitored rectal T during nighttime light exposure (7 out of the 20 reviewed), rectal T increased. On the other hand, the percentage of nighttime light interventions in HR studies was two times lower, possibly reducing the apparent effect of light on HR.

Three dichotomic variables were introduced into the analysis to check the influence of experimental conditions on light effect distribution: ‘Design’, ‘Night’, and ‘Light’. The ‘Design’ variable marked studies that had at least one of the following three limitations: un-counterbalanced crossover design [28,39,44] (two reported effects in [28]), a single measurement performed at the end of the light intervention [41], or a possible disappearance or emergence of the effect if the initial (pre-pulse) values of the measure in question at active and comparative lights had been adjusted to each other [53,60,61,76,84] (Table 1, Table 2 and Table 3). The ‘Night’ variable values were coded as ‘yes’ if the intervention light was used at night (at least partially within 00:00–06:00; 22 studies, 31%). The ‘Light’ variable values were coded as “big” or “not big” based on the difference between the intensities of active and comparative (dim) lights. The difference was classified as “big” if the light intensity was increased from a range between <1 and <90 lx for comparative light across a threshold 90–180 lx [79] to a lux value greater by at least 2 log units for active light. For example, a difference between 5 lx and 750 lx (used in [62]) was classified as “big” (lg 750—lg 5 = 2.2). According to this dual criterion, 29 studies (40%) used lights with a “big” difference in intensity between active and comparative lights. The statistics applied was a multinomial logistic regression analysis comprising light effect (classified as “−1”, “0”, or “1”) as the dependent variable, and ‘Design’, ‘Night’, and ‘Light’ altogether as independent variables. 

In fact, among all measures, rectal T was associated with the greatest percentage of studies that used nighttime and “big-difference” light (35% and 70%, respectively; Table 4), and both ‘Night’ and ‘Light’ factors were found to be significant regressors for the rectal T variable (according to multinomial logistic regression analysis; marked by asterisks in Table 4). In addition, factors ‘Night’ and ‘Design’ were significant for BP, and factor ‘Design’ was also significant for HRV (Table 4). Fewer energizing effects were associated with studies (1) having limitations in the design/analysis, (2) performed in daytime vs. night, and (3) for which the difference in intensity between active and comparative light was ‘not big’. Additionally, the studies differed in sample size (from 4 to 60 test participants).

To account for the influence of several variables at once on an ordinal variable, ancillary analysis was performed using general linear model (GLM) regression. This can provide the estimated mean of the light effects with a 95% confidential interval accounting for factor influence. The magnitude of the difference between the non-estimated mean and estimated mean can provide a rough assessment of the validity of the results of the non-parametric analysis. In particular, this would provide a more confident estimation of whether rectal T is more suitable for the detection of the energizing effect of light compared to HR. The dependent variable in the GLM analysis was weighted by study sample sizes; the sample size was halved in studies exploring between-subject design (*n* = 4) in order to be comparable to the number of test participants in studies with within-subject design.

Following GLM analysis, the mean rank value of the effects for rectal T diminished from 0.60 to 0.52. On the other hand, the mean rank value for HR increased from 0.27 to 0.42, and for skin T—from 0.13 to 0.35. Thus, even after accounting for sample size, ‘Design’, ‘Night’, and ‘Light’ factors, the estimated mean rank for rectal T remained the highest among all measures (Table 4). The confidence interval was above zero for rectal T and HR, confirming the results of the sign test. Confidence interval could not be computed for oxygen consumption (OC) and blood pressure (BP) because in all of the OC studies, light did not impact OC, while in BP studies the number of observations was not sufficient to perform three-factor analysis. 

Additional analysis revealed concordant changes during light interventions for rectal T and HR in seven studies in which they were measured simultaneously (tau = 0.69 and *p* = 0.066 via tau-b Kendall correlation). Other correlations were far from the significance threshold (including the correlation between HR and HRV: *n* = 17, *p* = 0.88) or could not be computed due to a low number of observations.

### 4.3. Measures Rating

Collectively, rectal T had the highest overall rank (Table 4, last column) among the measures based on having the greatest percentage of associated energizing effect (13 of 20 cases; 65%) and its significant preponderance over the opposite effect (13 cases vs. 1 case; *p* = 0.002). For HR, these two indices were 30% and *p* = 0.012. For other variables, they were ≤35% (except sympathetic nerve activity) and *p* > 0.1 (Table 4).

Based on these criteria, there is no sufficient evidence to deem 7 of the 14 analyzed physiological variables (oxygen consumption, skin conductance, blood pressure, heart rate variability, non-rectal inner temperature (digestive, tympanic, and oral T combined), skin temperature, and cortisol levels) to be reliable measures of the energizing effect of light (Table 4). Low ranking of blood pressure (BP) is confirmed with even greater certainty when two reported energizing effects due to BP are excluded from the analysis due to the fact that they cannot be classified as immediate (see explanation in Section 3.6).

There were four further measures for which the reviewed studies showed no evidence of energizing effect (skin blood flow, noradrenaline, salivary alpha-amylase, and thyroid-stimulating hormone levels). However, analysis of these four measures was limited due to insufficient data (only from one to three reported effects). No definite conclusion could be drawn for sympathetic nerve activity measured using microneurography (invasive); while the measure clearly reflected the energizing effect of light, it was used in only one study with only five test subjects.

Removal of the 11 light wavelengths studies from the analysis did not change the results significantly. Light wavelength studies included measurements of nine physiological variables, most often HRV, HR, and cortisol (*n* = 7, 6, and 4, respectively), while rectal T was not measured in those studies. For HR, the effects distribution slightly improved to 9/18/0 (*p* = 0.004), whereas the estimated mean effect slightly decreased to 0.39 (CI 0.04 ÷ 0.74). The overall suitability ratings for the measures remained the same.

## 5. Discussion

The results of the sign test, regression, and correlation analyses indicate that the energizing effect of light can be detected using rectal temperature and heart rate, and that their simultaneous measurement in a study may strengthen the reliability of the result. The review confirmed statistically, using cross-study analysis, that light causes greater effect on physiology when it is applied at night compared to daytime, and when the applied light is brighter. A stimulatory effect of light on core body temperature (rectal T) was observed not only for the nighttime light exposure, as was already known [3], but also for daytime exposure with notable consistency (46% of reported effects, more than for other analyzed measures). Interestingly, the effect of bright light exposure during daytime may emerge after (or be enhanced by) preliminary ingestion of melatonin (as shown by under-tongue and skin temperatures) [64]. This suggests that some physiological parameters (such as blood pressure) may be sensitive to melatonin secretion when reacting to bright light.

Unfortunately, measurement of rectal T is not as simple as measuring some other indices. Studies should consider certain requirements for (and limitations in) the use of rectal T to document the effects of light. Studies should ensure constant ambient temperature and constant posture of the subject 30–60 min prior to and during the intervention; significant effects take quite a long time to emerge—30–60 min (due to the inertia of the temperature regulation machinery), and the method may cause some inconvenience for the subject.

This review has certain limitations. It is possible that not all eligible studies were included, which may limit the accuracy of the review’s conclusions. The conclusions may also have changed if the review had been carried out on the subset of the studies reporting effect estimates (with variance) or *p*-values, which could form the basis of a more powerful analysis [94].

## 6. Conclusions

This review indicates that light causes an energizing effect in humans that can be objectively documented by measuring core (rectal) body temperature and additionally by measuring heart rate, which correlates with temperature (on a trend level). Further work should focus on acquiring more experimental data in humans with comparable experimental designs that allow extended statistical analyses and meta-analyses. The availability of objective measures opens up new avenues for combining psychometric estimates with core body temperature and heart rate in assessing the energizing effect of light.

## Figures and Tables

**Table 4:** Summary of the light effects (with “↑“ indicating energizing) in the reviewed studies.

Measure	N of Reports	Effect		Factor ^1^			Mean Effect		Overall Rating ^3^
		↑/ = /↓ N	↑ %	Design %	Night %	Light %	Mean	Estimated Mean (95% CI) ^2^	
1. Oxygen consumption (OC)	5	0/5/0	0	0	0	60	0.00	–	negative
2. Sympathetic nerve activity	1	1/0/0	100	0	0	0	1.00	–	likely s.
3. Skin conductance (SC)	6	1/5/0	17	0	0	0	0.17	0.18 (−0.26 ÷ 0.62)	negative
4. Skin blood flow (SBF)	3	0/3/0	0	0	0	33	0.00	–	unlikely s.
5. Noradrenaline (NA)	1	0/1/0	0	0	0	100	0.00	–	unlikely s.
6. Blood pressure (BP)	10	2/8/0	20	10 *	10 *	40	0.20	–	negative
7. Heart rate (HR)	33	10/22/1 *	30	3	18	30	0.27	0.42 (0.12÷0.72)	2
8. Heart rate variability (HRV)	20	7/8/5	35	15 *	20 ^(^*^)^	15	0.10	0.16 (−0.52 ÷ 0.85)	negative
9. Body temperature (T)									
Inner T:	34	17/13/4 **	50	12	11 *	65 ^(^*^)^	0.38	0.43 (0.13 ÷ 0.73)	–
− rectal T	20	13/6/1 **	65	10	35 ***	70 *	0.60	0.52 (0.25 ÷ 0.80)	1
− digestive, tympanic, oral T	14	4/7/3	29	14	29	57	0.07	0.13 (−0.42 ÷ 0.67)	negative
Skin T (including DPG)	15	4/9/2	27	13	7	60 ^(^*^)^	0.13	0.35 (−0.29 ÷ 0.99)	negative
10. Salivary alpha-amylase	3	0/3/0	0	0	67	0	0.00	–	unlikely s.
11. Cortisol	24	4/16/4	17	21	50	42	0.00	−0.01 (−0.25 ÷ 0.24)	negative
12. Thyroid-stimulating hormone (TSH)	3	0/3/0	0	33	67	0	0.00	–	unlikely s.

^1^ indicates columns in which a percentage of studies have a limitation in the design/analysis (‘Design’ column), studies were performed at night (‘Night’ column), and/or studies had a big difference in the light intensities between active and comparative lights (‘Light’ column); ^2^ estimated mean of the effect ranks (1—energizing, 0—neutral, −1—opposite to energizing) after accounting for the sample sizes (“weighted” mean) and the influence of the ‘Design’, ‘Night’, and ‘Light’ factors; ^3^ overall rank was assigned with regards to the suitability of the measure to document the energizing effect of light, the rank “unlikely s.” means “unlikely suitable” (for a detailed explanation, see Section 4.3). N—number; CI—confidence interval; DPG—distal-to-proximal gradient of skin T. ^(^*^)^ *p* < 0.1, * *p* < 0.05, ** *p* < 0.01, *** *p* < 0.001—significant distribution of the effects (one-sample sign test) or significance of the factor for effects distribution (according to multinomial logistic regression analysis).

## Supplementary Materials

The following supporting information can be downloaded at: https://www.mdpi.com/article/10.3390/clockssleep4040038/s1.
